# Supplementary material for: Patterns of Intron Gain and Loss in Fungi
Source: PLoS Biol. 2004 Nov 30;2(12):e422. doi: 10.1371/journal.pbio.0020422 (PMC532390; doi:10.1371/journal.pbio.0020422)
Supplement: Table S1 — Also available at http://genes.mit.edu/NielsenEtAl/. (4.3 MB ZIP). [file pbio.0020422.st001.zip › NielsenEtAl/html/787.html]

AN8843.1.NCU04277.1.MG09550.1.FG10211.1


```
 CLUSTAL W (1.82) Multiple Sequence Alignments - Introns Inserted


Sequence 1: NCU04277.1	358 aa
Sequence 2: FG10211.1	356 aa
Sequence 3: MG09550.1	356 aa
Sequence 4: AN8843.1	326 aa
Alignment Length: 364 aa
Number Identitical Residues: 216 aa
Alignment Score (without introns) 10024


MG09550.1 	----MASESFIIKTPSSSANIGPGFDVIGLALSLYLELHVTIDRSKGTSSSPLNCRVT~Y
NCU04277.1	MASSQQIEKFVIKTPCSSANIGPGFDVIGLALSMYLELHVTIDRSKTTSDKPLNCRIT~Y
FG10211.1 	------MESFIIKTPCSSANIGPGFDVIGLALSVYLELHVTIDRSKTKSELPLNCRIT~Y
AN8843.1  	----MSSKSFVIRTPCSSANIGPGFDVIGLALSLYLELQVTIDSSRTSSQQPLNCVIT2-
          	       :.*:*:**.*****************:****:**** *: .*. **** :*  

MG09550.1 	EGDGEESGDIPLNPDANLITRVALYVLRCHGQRAFPVETHVHVKNPIPLGRGLGSSGSAV
NCU04277.1	EGQGEE--DISLDPEVNLITRVALYVLRCNDQRSFPVETHVHIKNPIPLGRGLGSSGAAV
FG10211.1 	EGEGED--EISLDPEVNLLTRVALYVLRCNEQRSFPVETHIHIKNPIPLGRGLGSSGAAV
AN8843.1  	----------------------------CHDQRAFPVETRVHIVNPIPLGRGLGSSGTAV
          	                            *: **:*****::*: *************:**

MG09550.1 	VAGVMLGKMVGKLDHLDDNRLFDFCLMIE1RHPDNVGASLFGGFVGTYLNPLKPEDVART
NCU04277.1	VAGVQLGKEVGGLHDLTPERLFDYCLMIE1RHPDNVGAALFGGFVGTYLKPLTPEETART
FG10211.1 	VAGVALGKEVGGLKHLDNDRLFDYCLMIE1RHPDNVGAALYGGFVGTYLKPLTPEDVART
AN8843.1  	VAGVMLGNEVGQLG-LSKDRLLDYCLMIE1RHPDNVAASLFGGFVGTYLNELKPEDVARK
          	**** **: ** *  *  :**:*:***** ******.*:*:********: *.**:.**.

MG09550.1 	EIPLSEVLPAPAGGIDTGDTPPEPPHGIGHHIKFPWAPEIKAVAIIPNFEVATAKAREVL
NCU04277.1	EIPLSEVLPAPAGGVDTGVKPPSPPHGIGHHIKFPWAPEIKAVAIIPEFEVPTAKAREVL
FG10211.1 	EIPLSEVLPAPAGGEDTGKKPPNPPHGIGHHIKFPWAKEIKAVAIIPDFQVATHAARGVL
AN8843.1  	EIPLSEVLPAPAGGIDTGKRPPEPPLGIGHYRKFHWAKEIKAIAIIPDFVVPTANARNVL
          	************** ***  **.** ****: ** ** ****:****:* *.*  ** **

MG09550.1 	PAQYSRSDV0TFNLQRIALLPVALGQSPPDSGLIYLAMQDKLHQPYRQTLIPGLTDIVES
NCU04277.1	PAEYPRADV0TFNLQRIALLPVALGQSPPDPELINLAMQDKLHQPYRQVLIPGLTKIVES
FG10211.1 	PANYTRPDV0VFNLQRIALLPVALGQSPPDPELIHLAMQDKIHQPYRQTLIPGLTEIVET
AN8843.1  	PTSYSRADV0VFNLQRAALLPAALGSSPPDPDMIYLAMQDKVHQPYRQTLIPGLTEILRS
          	*:.*.*.** .***** ****.***.****. :* ******:******.******.*:.:

MG09550.1 	MTPTTQPGLLGVCLSGAGPTILALATDNFEQIAERIIAKFKTQN----ITCTWKLLEPAE
NCU04277.1	MTPATQPGLLGVCLSGAGPTILALATENFETIAQKIIEEFKSNN----IVCRWELLEPAD
FG10211.1 	MSPKTDEGFLGVCLSGAGPTILALATHNFEAIADKIIAKLREHNEKKDLPCQWKVLEPAE
AN8843.1  	MTPSTQPGLLGICLSGAGPTILALATERFSEIAELIIARFASQN----ISCKWKLLEPAH
          	*:* *: *:**:**************..*. **: ** .:  :*    : * *::****.

MG09550.1 	GTQVIRQ
NCU04277.1	GTTVTRA
FG10211.1 	GTHVIRS
AN8843.1  	DGAIVQR
          	.  : :
```
